# Synthesis and Exploration of Barium Stannate–Zirconate BaSn_1–*x*_Zr*_x_*O_3_ (0 ≤ *X* ≤ 1) Solid Solutions as Photocatalysts

**DOI:** 10.1021/acs.inorgchem.3c02874

**Published:** 2024-03-27

**Authors:** Tarek Alammar, Anja-Verena Mudring

**Affiliations:** † Department of Chemistry, College of Science, King Faisal University, P.O Box 400, Al-Ahsa 31982, Saudi Arabia; ‡ Department of Biological & Chemical Engineering, Intelligent Advanced Materials, Aarhus University, Aarhus 8200, Denmark; ⊥ Department of Physics, Umeå University, Umeå 901 87, Sweden

## Abstract

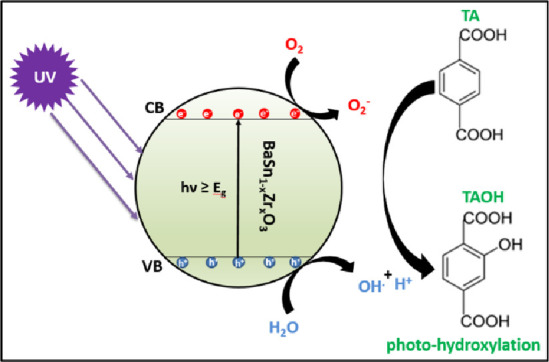

Employing ionic liquid-assisted microwave synthesis and moderate heat treatment allows for the preparation of otherwise difficult-to-obtain perovskite-type BaSn_1–*x*_Zr*_x_*O_3_ solid solutions (0 ≤ *x* ≤ 1). The impact of substituting Sn for the crystal structure, crystallinity, morphology, and photocatalytic performance was investigated. The obtained materials are characterized by X-ray diffraction, scanning electron microscopy, Brunauer–Emmett–Teller (BET) surface area analysis, X-ray photoelectron spectroscopy, UV–Vis diffuse reflectance spectroscopy, photoluminescence spectroscopy, and Raman and IR spectroscopy. SEM images show that the morphology of the samples varies from rods for *x* = 0, 0.2 to spherical for *x* = 0.5, 0.8, 1. Upon Zr for Sn substitution, the band gap changes from 3.1 to 5.0 eV as the valence and conduction bands move to lower and higher energies. The photocatalytic activities of the BaSn_1–*x*_Zr*_x_*O_3_ samples in the hydroxylation of terephthalic acid (TA) follow the order BaSn_0.5_Zr_0.5_O_3_> BaSn_0.8_Zr_0.2_O_3_> BaSnO_3_> BaSn_0.2_Zr_0.8_O_3_> BaZrO_3_. The superior photocatalytic activity of BaSn_0.5_Zr_0.5_O_3_ can be attributed to the synergistically favorable combination of a suitable band structure, band gap size, and increased surface area-to-volume ratio, resulting in a diminished crystalline particle size unattainable from samples prepared via traditional synthetic routes or without ionic liquid.

## Abstract 2

Ionic liquid-assisted microwave synthesis withmoderate heat treatment allows for the preparation of otherwise difficult-to-obtain perovskite-type BaSn_1_-_*x*_Zr_*x*_O_3_ solid solutions. Amongst those, BaSn_0.5_Zr_0.5_O_3_ shows superior photocatalytic activity in the photohydroxylation of terephtalicacid, which originates from a synergistically favorable combination of bandstructure and gap as well as particle size and surface unattainable fromsamples prepared via traditional synthetic routes or without ionic liquid.

## Introduction

Perovskite-type stannate and zirconates are functional materials that are attractive for various applications because of their physical–chemical properties, such as a high chemical and thermal robustness, high melting point, high dielectric constant, low thermal expansion coefficient, and appreciable mechanical strength.^1^ These materials have been useful for applications in electro-optic devices, fuel cells and proton conductors, relaxor ferroelectrics, and dielectric materials for use in capacitors, Li-ion batteries, chemical sensors, superconductors, and for photocatalysis.^2,3^

Traditionally, solid-state synthesis is the traditional route for the preparation of these compounds. They required a high reaction temperature (1100–1400 °C), prolonged reaction time (many hours), and some additives as flux. However, this approach leads typically to products with low surface area and broad particle size distribution and often impurities originating from unreacted starting materials or unwanted oxidic side products,^4,5^ which is particularly problematic if the product is used as a catalyst. For that reason, wet chemical methods, such as the hydrothermal method,^6^ sol–gel synthesis,^7^ flash pyrolysis,^8^ and the Pecchini method,^9^ have been explored for overcoming these shortcomings. In the context of low temperature and green synthesis of the materials, microwave synthesis comes into focus as it is an efficient technique owing to the low energy consumption, comparatively low overall reaction temperature, short reaction time, and higher reaction rate and yield, which also promotes the formation of nanoparticles. Moreover, microwave radiation can be efficiently absorbed by ionic liquids (ILs) as a result of their unique salt-like properties, which gives them a particular advantage.^10^ ILs have attracted attention for the nanosynthesis of materials as they can efficiently affect the growth of (nano-) particles by electronic and steric interactions.^11,12^ They can serve as the reaction medium, microwave receptor, template, structure-directing agent, and surface protector all in one, as we reported on the preparation of quasi-binary Ce_0.5_M_0.5_O_2_ (*M* = Ti, Zr, Hf) solid solutions.^13^ Through the ionic liquid-assisted microwave, we managed to obtain solid solutions of the Sr_1–*x*_Ba_*x*_SnO_3_ (x = 0, 0.2, 0.4, 0.8, 1) perovskite series at low temperature.^14^ This prompted us to explore whether this method can be extended to prepare more compositionally complex quaternary BaSn_1–*x*_Zr*_x_*O_3_ (0 ≤ *x* ≤ 1) solid solutions. Albeit there is strong interest from a materials and properties viewpoint in barium stannate–zirconate solid solutions, reports on their preparation are virtually nonexistent, presumably because of the difficulty in synthesis. This prompted us to carry out such investigations and, in particular, explore the ionic liquid-based microwave-assisted method for the synthesis of BaZr_1–*x*_Sn_*x*_O_3_ solid solutions and further explore the effect of Zr for Sn substitution on the structure, morphology, optical absorption, energy-band structures, and photocatalytic properties systematically.

## Experimental Section

### Materials

Chemicals were purchased from Iolitec (lithium bis(trifluoromethansulfonyl)amide 99%), Sigma-Aldrich (tin(VI)chloride pentahydrate 98%, 1-methylimidazole 99%, ethanol (p.a.), 1-chlorobutane 99%), Fisher Scientific (sodium hydroxide 98%), J.T. Baker (acetonitrile 99.5%, ethyl acetate 99%), and Alfa Aeaser (barium acetate 99%, terephthalic acid 98%). The reagents were directly used without further purification. The ionic liquid 1-butyl-3-methylimidazolium bis(trifluoromethanesulfonyl)amid, [C_4_mim][Tf_2_N], was prepared according to a modified literature procedure.^15^

### Synthesis

For the synthesis of BaZr_1–*x*_Sn*_x_*O_3_ samples, the required stoichiometric amounts of Ba(CH_3_COO)_2_, SnCl_4_·5H_2_O, and ZrCl_4_ to give about 100 mg final product and 0.15 g finely ground NaOH powder were added to a mixture made of 1 mL deionized water and 2 mL ionic liquid ([C_4_mim][Tf_2_N]). The reaction mixture was stirred vigorously for 30 min, and then irradiated with a single-mode microwave operating at 2455 MHz (CEM Discover, Kamp-Lintfort, D) in a 10 mL glass vessel equipped with a Teflon© septum for 10 min at 85 °C. The product was separated by centrifugation, washed thoroughly with ethanol and distilled water, and dried overnight in air at 80 °C. The dried product was calcined at 700 °C in air for 3 h.

### Characterization

Powder X-ray diffraction measurements were carried out on a PANalytical powder diffractometer with an Xcelerator detector employing CuKα radiation (λ = 0.15406 nm).

Scanning electron microscopy (SEM) measurements were performed with a high-resolution thermally aided field SEM (Zeiss, LEO 1530 Gemini) with a field emission gun (FEG) and an acceleration voltage of *U*_acc_ = 0.2–30 kV. For the SEM measurements, the BaSn_1–*x*_Zr_*x*_O_3_ powders were dispersed on a carbon film and dried under vacuum for 20 min.

X-ray photoelectron spectroscopy (XPS) measurements were undertaken on a physical electronic 5500 multitechnique system with a standard aluminum source. The analysis spot size was 1 × 1 mm. Samples were mounted on double-sided scotch tape. The binding energies in the XPS spectra are calibrated against the C 1s signal (284.8 eV), corresponding to adventitious physisorbed carbon oxide.

Attenuated total reflection (ATR) spectroscopy was carried out on an Agilent Technologies Cary 630 FT-IR spectrometer equipped with a diamond crystal. Solid samples were pressed onto the crystal.

Raman spectra were obtained at 150 mW using a Horiba Xplora Raman microscope (Horiba Scientific) at room temperature. Laser irradiation with 785 nm light was used for excitation. Silicon was used to calibrate the Raman shifts.

UV–vis spectra were measured at room temperature using an Agilent Cary 60 spectrometer with a dip probe coupler and a VideoBarrelino (Harrick) in reflection mode. To determine the optical absorption properties of the BaSn_1–*x*_Zr*_x_*O_3_ samples, the respective absorption edges were obtained through linear extrapolation of the steep part of the UV absorption toward the baseline. Tauc plots were used to calculate the optical band gaps (*E*_*g*_) according to (α*h*ν)^n^ = *B*(*h*ν-*E*_*g*_), where *h*ν is the photon energy, α the absorption coefficient, *B* is related to the effective masses associated with the valence and conduction bands, and *n* is either 2 for an indirect allowed transition or 1/2 for the direct allowed transition. Plotting (α*h*ν)^2^ versus *h*ν based on the spectral response gives the extrapolated corresponding *E*_*g*_ values. The band edge position was obtained according to *E*_*c*_ = *X* – *E*_*0*_ – 0.5 *E*_*g*_, where *E_c_* is the conduction band (CB) edge potential, *X* is the geometric mean of the Sanderson electronegativity of the constituent atoms, *E*_*g*_ is the band gap energy, and *E*_*0*_ is the scale factor relating the reference electrode redox level to the absolute vacuum level (*E*_*0*_ = 4.5 eV for a normal hydrogen electrode (NHE)).

Brunauer–Emmett–Teller (BET) surface area nitrogen physisorption experiments were carried out in a modified Autosorb 1C setup (Quantachrome). The sample was thermally pretreated at 200 °C for 2 h in He. The physisorption measurements were performed at the boiling point of liquid N_2_ (78 K). The surface area is calculated according to the BET (Brunauer–Emmett–Teller) equation.

PL (photoluminescence) spectra were measured on an Agilent Technologies Cary Eclipse fluorescence spectrophotometer equipped with a Xenon flash lamp and built-in excitation and emission filters. For the measurement, liquid samples were filled into a standard 10 mm quartz cuvette and positioned in the incoming beam of the sample chamber.

Photohydroxylation of terephthalic acid (TA) was performed in a reactor, which contained a suspension of 100 mg of photocatalyst in 100 mL of 0.01 M NaOH solution containing 3 mM terephthalic acid (C_8_H_6_O_4_). The suspension was continuously stirred in the dark for 30 min to establish an adsorption/desorption equilibrium, and then illuminated using a 100 W Xe arc lamp (Newport Oriel Instruments). The lamp was switched on 30 min prior to the illumination of the samples in order to stabilize the power of its emission at λ > 320 nm (a cutoff filter FSQ-WG320 was used to eliminate most of the spectra <320 nm). Every 30 min, about 3 mL aliquots were sampled and filtered by nylon syringe filters (pore size 0.2 μm) to remove the photocatalyst before analysis by fluorescence spectrometry (Agilent Technologies Cary Eclipse Fluorescence Spectrophotometer) at 426 nm. Photogenerated holes in the catalyst generate ·OH radicals from surface-absorbed water, which decompose terephthalic acid to 2-hydroxy terephthalic acid, which exhibits a typical fluorescence band at 426 nm when excited at a wavelength of 320 nm. Consequently, an increase in photoluminescence intensity corresponds to a growing amount of photogenerated ·OH radicals.

## Results and Discussion

**Figure 1:**
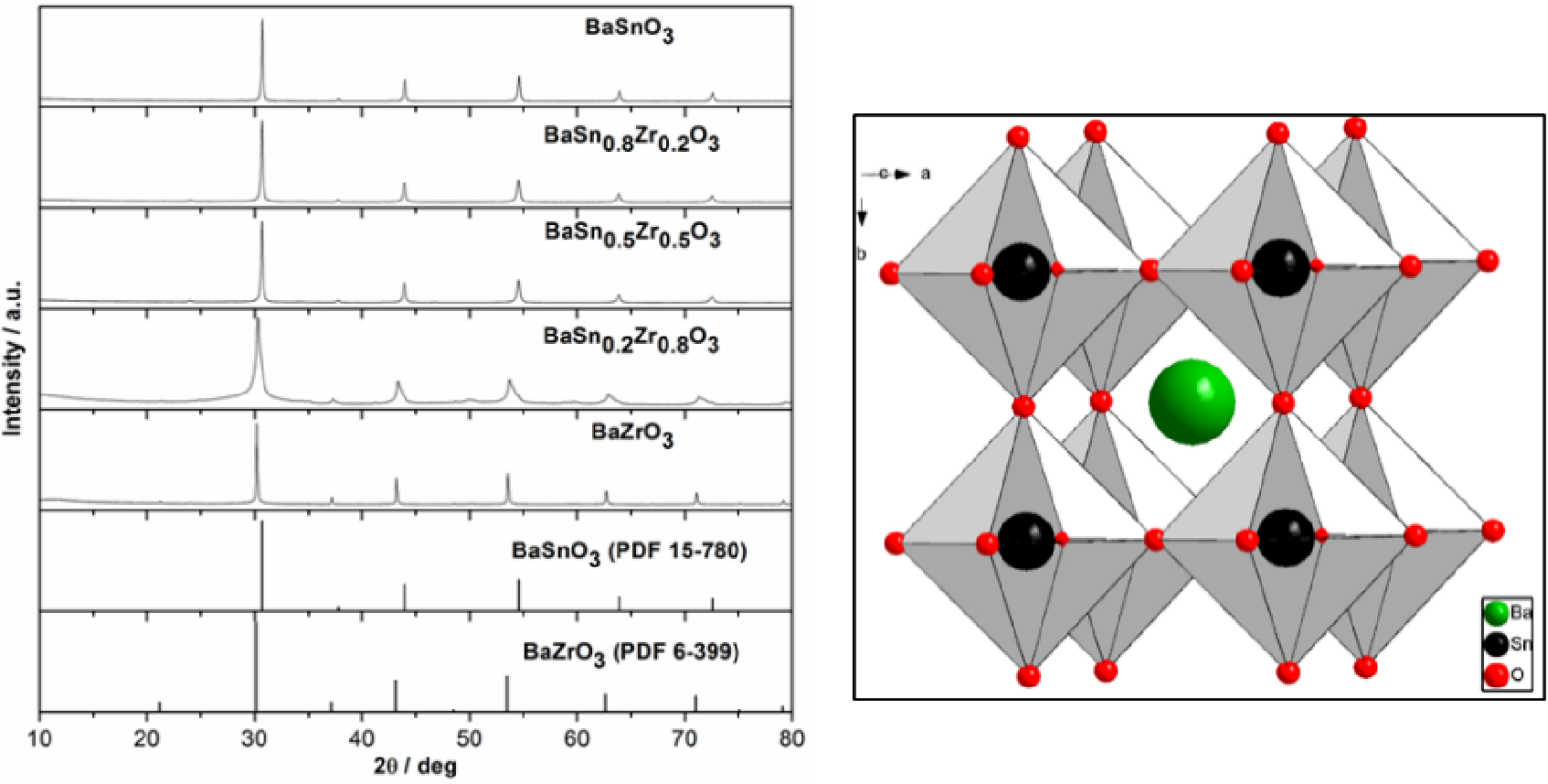
PXRD patterns of BaSn_1–*x*_Zr*_x_*O_3_ nanopowders in comparison with the database patterns of BaZrO_3_ (PDF 6–399) and BaSnO_3_ (PDF 15–780) (top), and representation of the BaSnO_3_ crystal structure (bottom).

Both BaSnO_3_ and BaZrO_3_ adopt a cubic perovskite type of structure. The powder X-ray diffraction (PXRD) patterns of the obtained BaZr_1–*x*_Sn*_x_*O_3_ (0 ≤ *x* ≤ 1) solid solution samples and reference data for BaSnO_3_ (PDF 15–780) and BaZrO_3_ (PDF 6–399) are compared in Figure 1. The X-ray diffraction patterns of the end phases of the solid solution, BaSnO_3_ and BaZrO_3_, can be indexed to the cubic perovskite structure (Supporting Information, SI-1, for full details on Rietveld refinements for all samples). No traces of impurities that potentially could be binary oxides, such as ZrO_2_, BaO, or SnO_2_, carbonate, or hydroxides, are observed. Furthermore, the PXRD patterns reveal that with increasing Zr concentration, the position of all corresponding diffraction peaks shift toward lower diffraction angles (2θ) (Figure 1). This trend originates from the fact that the ionic radius of Sn^4+^ at 71 pm is smaller than that of Zr^4+^ at 80 pm.^16^ It is noteworthy that the diffraction peaks are symmetric, underlining that indeed true solid solution formation took place and not physical mixtures of BaSnO_3_ and BaZrO_3_ were obtained. The average crystallite sizes were estimated from the fwhm values of the most intense diffraction peaks using the Debye–Scherrer equation and are summarized in Table 1. The determined crystallite sizes range from 19.86(1) nm for BaSn_0.2_Zr_0.8_O_3_ to 79.04(1) nm for BaZrO_3_.

**Table 1:** Lattice Parameters, Crystallite Size, and Crystallite Strain for the BaSn_1–*x*_Zr_*x*_O_3_ Samples as Determined from the PXRD Data

sample	*a*/Å	crystallite size (nm)	crystallite strain (ε_str_)/10^–3^
BaSnO_3_	4.11764(7)	53	0.67
BaSn_0.8_Zr_0.2_O_3_	4.11947(7)	40	1.65
BaSn_0.5_Zr_0.5_O_3_	4.11872(1)	40	1.40
BaSn_0.2_Zr_0.8_O_3_	4.17007(2)	20	3.80
BaZrO_3_	4.18794(7)	79	0.85

**Figure 2:**
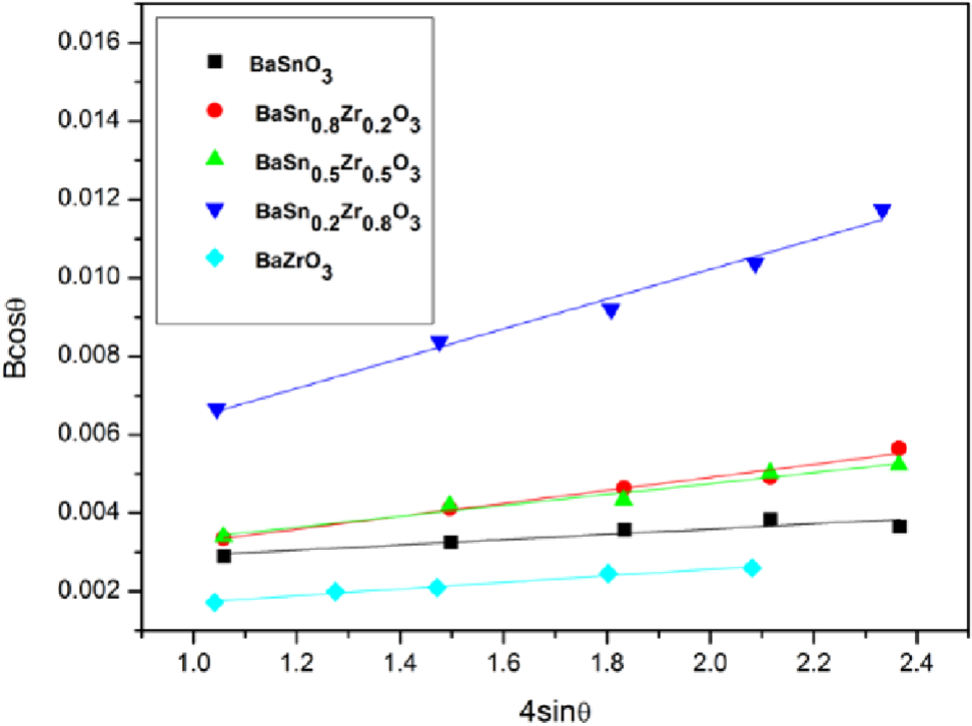
Williamson–Hall plots of BaSn_1–*x*_Zr*_x_*O_3_.

The crystallite strain of the BaSn_1–*x*_Zr*_x_*O_3_ samples was assessed using the Williamson–Hall (W–H) equation.^17^ The plot of *B_hkl_*cos θ against 4sin θ for the synthesized BaSn_1–*x*_Zr*_x_*O_3_ samples is shown in Figure 2. According to this method, the slope of the linear fit gives strain ε. The obtained values are summarized in Table 1. The crystallite strain values ranged from 0.00084 for BaZrO_3_ to 0.0038 for BaSn_0.2_Zr_0.8_O_3_. Furthermore, the small strain values point to the existence of only a few lattice defects and high sample crystallinity. Among all samples, BaZrO_3_ shows the highest crystallinity, while BaSn_0.2_Zr_0.8_O_3_ is the least crystalline material.

**Figure 3:**
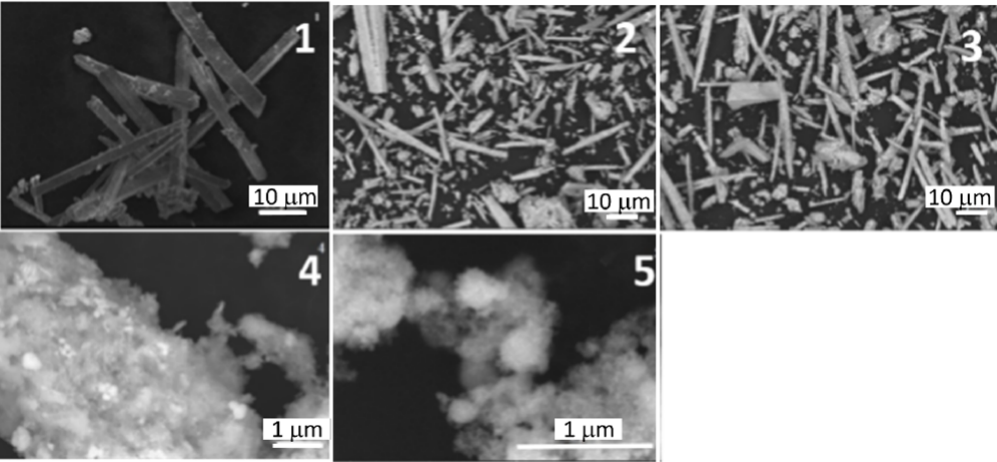
SEM images of BaSn_1–*x*_Zr*_x_*O_3_. (1) BaSnO_3_, (2) BaSn_0.8_Zr_0.2_O_3_, (3) BaSn_0.5_Zr_0.5_O_3_, BaSn_0.2_Zr_0.8_O_3_ (4), and BaZrO_3_ (5).

The impact of the Zr^4+^ substitution for Sn^4+^ on the sample morphology was examined by electron microscopy. As shown in Figure 3, an evolution of rods to more spherical particles is observed with increasing Zr content in the lattice of BaSnO_3_. Spherical particles were observed for BaZrO_3_ and for BaSn_0.8_Zr_0.2_O_3_ with sizes varying from 50 to 500 nm. It is remarkable that the BaSn_0.5_Zr_0.5_O_3_, BaSn_0.2_Zr_0.8_O_3_, and BaSnO_3_ samples show similar morphology (Figure 3, top row). They consist of rod-shaped particles with diameters in the range of 1–5 μm and a length of several micrometers. The rods are less homogeneous for BaSn_0.5_Zr_0.5_O_3_ and BaSn_0.2_Zr_0.8_O_3_ in comparison with BaSnO_3_.

**Figure 4:**
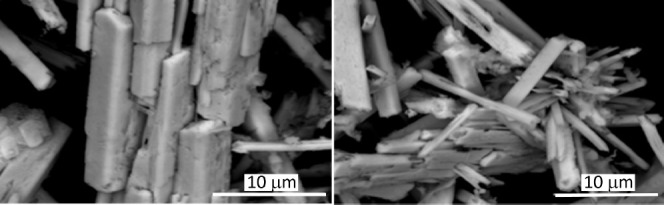
SEM images of the BaSnO_3_ sample prepared in demineralized water without an ionic liquid (scale bar 10 μm).

Moreover, to demonstrate the influence of IL on the morphology and structure of BaSnO_3_, BaSnO_3_ was prepared in demineralized water without IL by using otherwise the same reaction conditions. It can be clearly seen from the SEM images revealed in Figure 4 that the sample mostly shows fairly large, irregular agglomerates composed of rod-shaped particles with a wide size distribution. Both, in length and diameter, the rods are larger than those for material formed in the presence of IL. Figure 4 suggests that the rod-like particles preferred to self-assemble and grow radically from the center. As no template or surfactant was used in this synthesis, the decrease in surface energy is expected to be the dominant factor in controlling the shape of particles. The XRD pattern (Supporting Information, SI-2) reveals further that not only perovskite-type BaSnO_3_ is formed during the synthesis but also BaCO_3_ (PDF 5–378). Thus, it can be concluded that the IL not only helps to obtain nanoparticulate material with defined morphology but also allows to obtain a phase-pure product.

**Figure 5:**
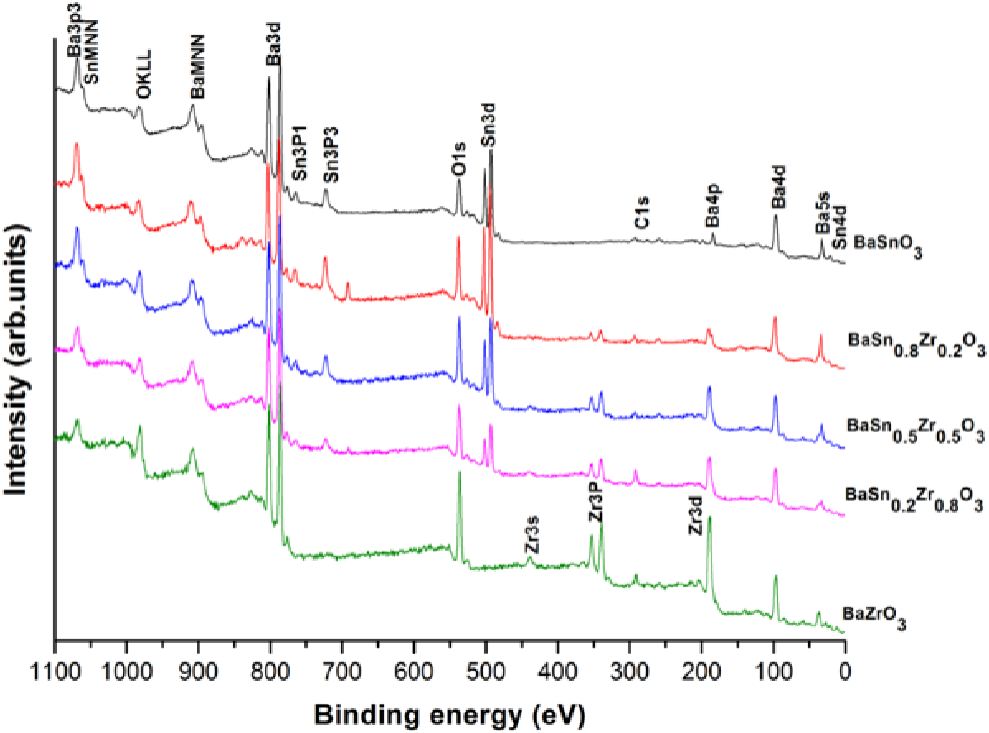
X-ray photoelectron spectrum survey scans of the BaSn_1–*x*_Zr*_x_*O_3_ samples.

The elemental composition of the BaSn_1–*x*_Zr_*x*_O_3_ solid solutions and the oxidation state of each element were probed by X-ray photoelectron spectroscopy (XPS). The XPS survey spectra of the BaSn_1–*x*_Zr*_x_*O_3_ solid solution samples are presented in Figure 5, revealing Ba, Sn, Zr, and O. The binding energies of the elements on the surface of the samples are summarized in Table 2. SI-3 shows the region scans for Ba 3d, Sn 3d, Zr 3d, and O 1s of all samples. Fitting XPS spectra for Ba 3d in BaSn_1–*x*_Zr_*x*_O_3_ uncovers two overlapping peaks, which can be assigned to Ba 3d5/2 at 797.1–1797.7 eV and Ba 3d3/2 at 794.4–795.1 eV with a peak separation of 15.3 eV, verifying the Ba^2+^.^18^ For Sn 3d in the BaSn_1–*x*_Zr_*x*_O_3_ samples, the XPS spectra show two peaks at 485.5–486.1 eV and 494.1–494.5 eV with a peak separation of 8.5 eV, which originate from Sn 3d_5/2_ and Sn 3d_3/2_, respectively. The peak separation of 8.5 eV matches that reported for Sn 3d in SnO_2_.^19^ Consequently, an oxidation state of +IV can be assigned to the Sn species on the samples surface. The Sn 3d peaks show a chemical shift to higher binding energy with increasing Zr content in the BaSnO_3_ lattice. The change in binding energy is due to the different ionic radii of the Sn^4+^ and Zr^4+^ cations, where the ionic size of Zr^4+^ is slightly larger than that of Sn^4+^, resulting in an increased metal–oxygen distance. The Zr 3d spectra show two peaks at 183.7–184.1 eV for Zr 3d_5/2_ and 181.2–181.9 eV for 3d_3/2_. The peak separation of Zr 3d (∼2.3 eV) is in agreement with the magnitude of the spin–orbit splitting constant of Zr 3d reported for ZrO_2_, hence, the oxidation state of zirconium is confirmed to be +IV. As shown in Supporting Information, SI-3, the O 1*s* spectra show broad, asymmetric bands that can be fitted to two peaks with binding energies of 529.1–529.8 eV and 530.9–531.7 eV. The peak at 529.1–529.7 eV belongs to the O_2_-lattice oxygen. The lower intensity peak at 530.9–531.7 eV is attributed to surface adsorbed OH^–^. The binding energy values are similar to those previously reported for BaSnO_3_ and BaZrO_3_.^20,21^ No peaks that would point to impurities or contamination from the employed ionic liquid could be detected. The XPS results confirm the formation of a single pure phase of the samples consistent with XRD.

**Table 2:** XPS Binding Energies (±0.1) in eV of Sn, Zr, O, and Ba in BaSn_1–*x*_Zr_*x*_O_3_ Samples

element	BaSnO_3_	BaSn_0.8_Zr_0.2_O_3_	BaSn_0.5_Zr_0.5_O_3_	BaSn_0.2_Zr_0.8_O_3_	BaZrO_3_
Sn (3d_5/2_; 3d_3/2_)	485.5; 494,1	485.9; 494.5	486.2; 494.5	486.1; 494.5	–
Zr (3d_5/2_; 3d_3/2_)	–	184.1; 181.9	183.7; 181.2	183.8; 181.5	183.8; 181.6
O (1*s*)	529.1; 530.9	529.8; 531.5	529.6; 531.7	529.5; 531.5	529.5; 531.5
Ba (3d_5/2_; 3d_3/2_)	779.1; 794.4	779.7; 795.1	779.6; 795.0	779.7; 795.0	779.5; 794.8

**Figure 6:**
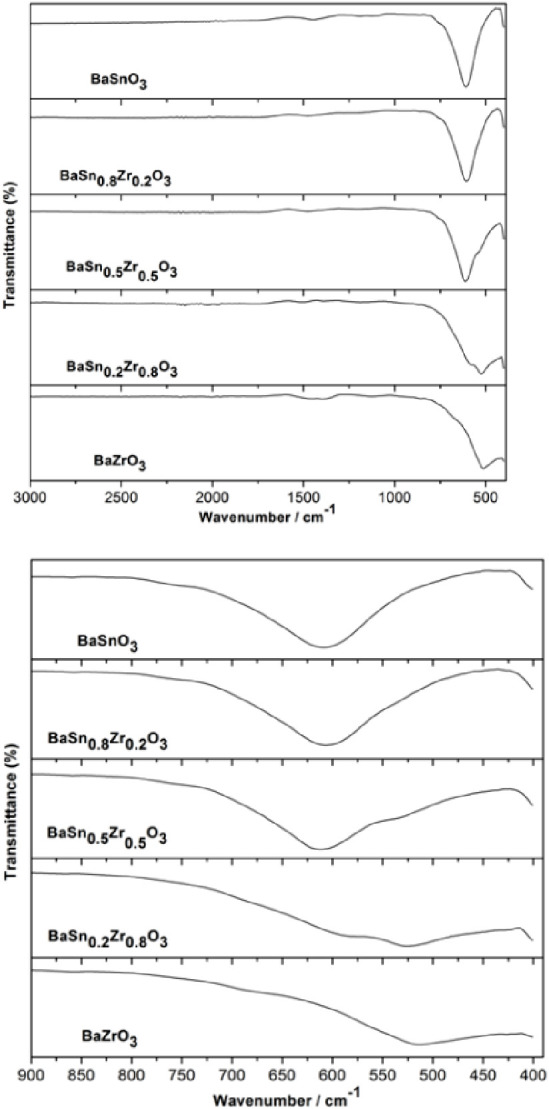
(Top) IR spectra of BaSn_1–*x*_Zr_*x*_O_3_ samples and (bottom) enlarged view in wavenumber 500–900 cm^–1^.

IR spectra of BaSnO_3_, BaZrO_3_, and their solid-solution BaSn_1–*x*_Zr*_x_*O_3_ samples in the region from 390 to 3000 cm^–1^ are shown in Figure 6. BaSnO_3_ shows an absorption band in the range 467–800 cm^–1^, with a center of 612 cm^–1^. This intense stretching vibration band can be attributed to the Sn–O vibration in the stannate group {SnO_6_}.^22^ The band center in BaZrO_3_ is located at 519 cm^–1^. This band is due to the Zr–O vibration in the zirconate group {ZrO_6_}, which appears to be slightly broader in comparison to the corresponding Sn–O band. When Zr^4+^ replaces Sn^4+^ in the BaSn_1–*x*_Zr*_x_*O_3_ solid solution samples, two bands, which individually can be assigned to Sn–O and Zr–O can be seen. Furthermore, the Sn–O band shows a blue shift, while the Zr–O shows a red shift with increasing Zr content in the lattice of BaSnO_3_ (see the Figure 6(bottom)). This can be attributed to Zr^4+^ being larger than Sn^4+^ in ionic radius, so that the Zr–O distance will be shorter than that of Sn–O. Thus, the shoulder on the low-frequency side of band Sn–O in BaSn_0.5_Zr_0.5_O_3_ is attributed to Zr–O and the shoulder on the higher frequency side of Zr–O in the Zr^4+^-richer sample (BaSn_0.2_Zr_0.8_O_3_) can be assigned to Sn–O vibration.

The Raman spectra of BaSn_1–*x*_Zr*_x_*O_3_ samples are given in the Supporting Information SI-4. Based on group theory, no first-order Raman active modes would be expected for materials crystallizing with the *Pm*-3m space group. As expected, the spectra of samples show only featureless, broad, very low intensity bands that can originate from strain, grain boundaries, and oxygen vacancies, which can locally lower the internal symmetry leading to activate the Raman-forbidden modes.^23^ Dhahri et al. reported that at a short- range between 100 and 740 cm^–1^, the BaZrO_3_ with cubic symmetry is polarized, where this short-range can be linked to the distorted ZrO_6_ clusters.^24^ Cerda et al. attributed the observed broad bands for BaSnO_3_ to the presence of local defects.^25^

**Figure 7:**
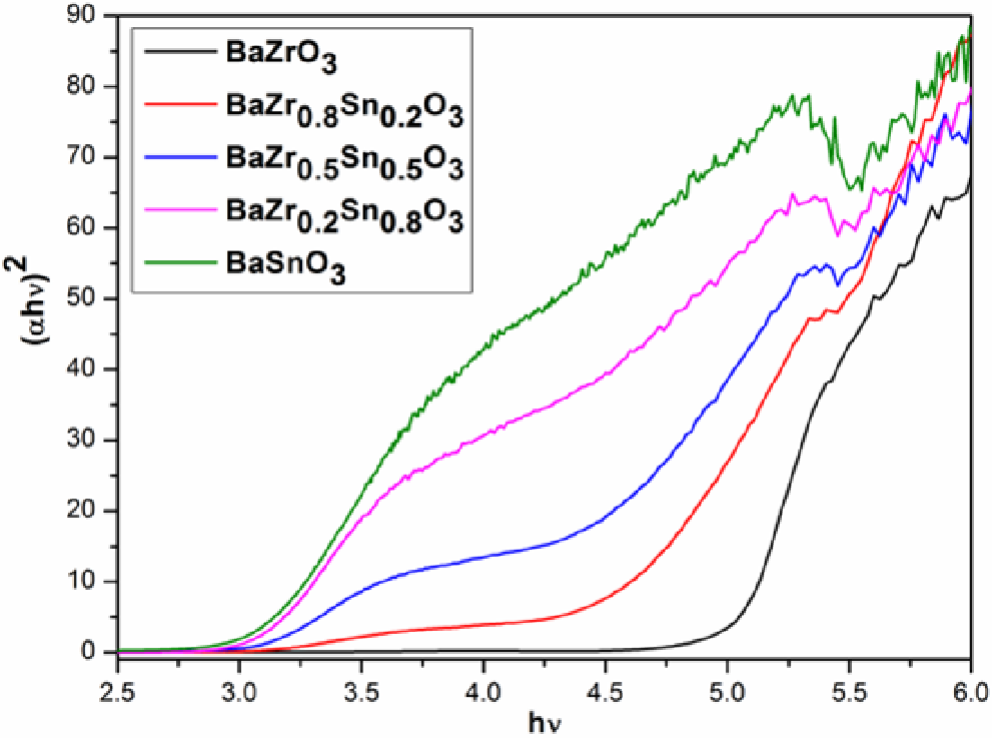
(α*h*ν)^2^–*h*ν curves of the BaSn_1–*x*_Zr_*x*_O_3_ samples.

UV–visible diffuse reflectance spectroscopy (DRS) was employed to determine the optical absorption properties of the BaSn_1–*x*_Zr*_x_*O_3_ samples. The respective absorption edges were determined through the linear extrapolation of the steep part of the UV absorption toward the baseline. Supporting Information SI-5 shows the DRS spectra of the BaSn_1–*x*_Zr_*x*_O_3_ samples. The UV–vis spectrum of BaSnO_3_ shows an absorption edge at 430 nm, which originates from the transition of O 2p electrons of the valence band (VB) into Sn 5s states of the conduction band (CB).^9^ BaZrO_3_ reveals an absorption edge at 255 nm, which corresponds to the transition of O2*p* electrons into Zr 4d levels.^26^ When Zr^4+^ replaces Sn^4+^ in the BaSn_1–*x*_Zr*_x_*O_3_ solid solution, the absorption edge shifts toward shorter wavelengths. As shown in Figure 7 and Table 3, the band gaps of the BaSn_1–*x*_Zr_*x*_O_3_ samples vary from 3.1 eV for BaSnO_3_ to 5.02 eV for BaZrO_3_, which is expected as the transition occurs from the filled oxygen levels to the empty metal states. As the Sn 5s levels are at lower energies compared to the Zr 4d, BaSnO_3_ is expected to have a lower band gap compared to BaZrO_3_, and the band gap will increase with increasing Zr content in the lattice of BaSnO_3n_. A similar observation was made for BaZr_1–*x*_Ta_*x*_O_3_ (*x* = 0 to 0.04) solid solutions.^27^

**Table 3:** Band Gap, Electronegativity, and Band Edge Position for BaSn_1–*x*_Zr_*x*_O_3_

sample	band gap *E*_*g*_/eV	electronegativity/eV	*E_c_* (NHE) /eV	*E_v_* /eV
BaSnO_3_	3.1 ± 0. 2	5.460	–0.59 ± 0.04	2.51 ± 0.04
BaSn_0.8_Zr_0.2_O_3_	3.1 ± 0.2	5.421	–0.64 ± 0.04	2.47 ± 0.04
BaSn_0.5_Zr_0.5_O_3_	4.2 ± 0.2	5.362	–1.24 ± 0.04	2.96 ± 0.04
BaSn_0.2_Zr_0.8_O_3_	4.5 ± 0.2	5.304	–1.45 ± 0.04	3.05 ± 0.04
BaZrO_3_	5.0 ± 0.2	5.266	–1.74 ± 0.04	3.28 ± 0.04

The bottom CB level and top VB level values of the BaSn_1–*x*_Zr_*x*_O_3_ samples, calculated according to Butler and Ginley,^28^ are summarized in Table 3. They vary from −1.74 eV for BaZrO_3_ to −0.59 eV for BaSnO_3_, and the valence band (VB) edge potentials range from 3.28 eV for BaZrO_3_ to 2.51 eV for BaSnO_3_. Moreover, the bottom level of the conduction band for all samples is much more negative than the reduction potential of *E*° (O_2_/·O^–^_2_)(−0.33 V vs NHE), while the top level of the valence band (EVB) of all samples is more positive than the oxidation potential of the *E*° (·OH/H_2_O) (2.3 vs NHE). Hence, the results indicate that the band edge positions of all BaSn_1–*x*_Zr_*x*_O_3_ samples satisfy the electrochemical requirements for the hydroxylation of terephthalic acid (TA), and that the BaSn_1–*x*_Zr_*x*_O_3_ samples might be able to catalyze the hydroxylation of terephthalic acid to hydroxyterephthalic acid (TAOH) under UV irradiation.

**Figure 8:**
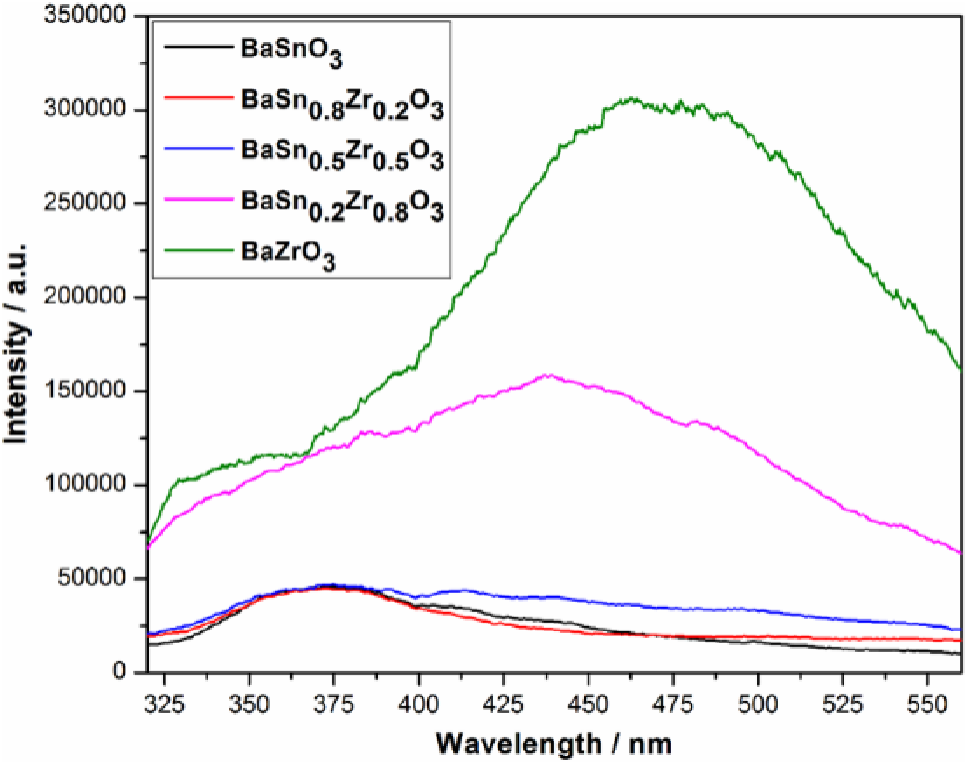
Photoluminescence spectra of the BaSn_1–*x*_Zr*_x_*O_3_ samples (excitation wavelength: 290 nm).

PL spectra of the BaSn_1–*x*_Zr_*x*_O_3_ samples were measured to obtain information regarding the recombination rate of charge carriers, where a high rate of charge carriers recombination is associated with PL spectrum with high intensity.^29^ As shown in Figure 8, the BaSnO_3_, BaSn_0.8_Zr_0.2_O_3_, and BaSn_0.5_Zr_0.5_O_3_ samples reveal the lowest PL intensities, indicating a low rate of charge carrier recombination, which lets expect an enhanced photocatalytic performance compared to other samples with stronger PL intensities.

**Figure 9:**
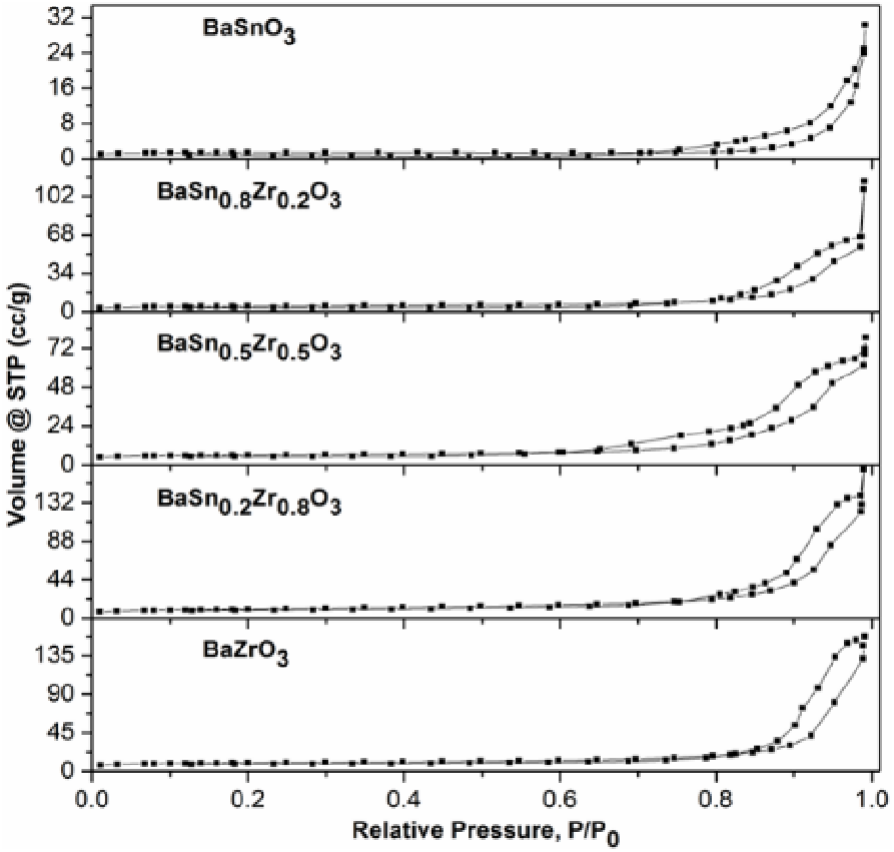
N_2_ adsorption–desorption isotherms of the BaSn_1–*x*_Zr*_x_*O_3_ samples.

The isotherms for the nitrogen adsorption–desorption experiments of the respective BaSn_1–*x*_Zr*_x_*O_3_ samples (Figure 9) are type IV with a hysteresis loop, which is characteristic of mesoporous materials. The pore size distributions of the as-prepared samples were determined by the BJH method from the adsorption branch. The pore size values ranged from 16.66 nm for BaSnO_3_ to 30.38 nm for BaSn_0.2_Zr_0.8_O_3_. The BET-specific surface area, pore volumes, and pore diameters of the samples are summarized in Table 4. The porosity can be attributed to the interparticle voids present in the respective BaSn_1–*x*_Zr*_x_*O_3_ samples. Moreover, the specific surface area values vary from 4 m^2^ g^–1^ for BaSnO_3_ to 36.37 m^2^ g^–1^ for BaSn_0.2_Zr_0.8_O_3_. It can be noticed that the specific surface area increases with an increase in the Zr content in the BaSnO_3_ lattice. A comparison with the SEM images (Figure 3) confirms that.

**Table 4:** BET Surface Areas, Pore Diameters, and Pore Volumes of the BaSn_1–*x*_Zr*_x_*O_3_ Samples

sample	BET surface area SBET (m^2^/g)	pore diameter (nm)	nitrogen pore volume, *V*_pore_ (cm^3^/g)
BaSnO_3_	4.82	16.66	0.019
BaSn_0.8_Zr_0.2_O_3_	17.74	20.02	0.088
BaSn_0.5_Zr_0.5_O_3_	21.52	17.94	0.095
BaSn_0.2_Zr_0.8_O_3_	36.37	30.38	0.187
BaZrO_3_	34.44	23.83	0.203

### Evaluation of Photocatalytic Activity of BaSn_1–*x*_Zr*_x_*O_3_ in the Photohydroxylation of Terephthalic Acid

**Figure 10:**
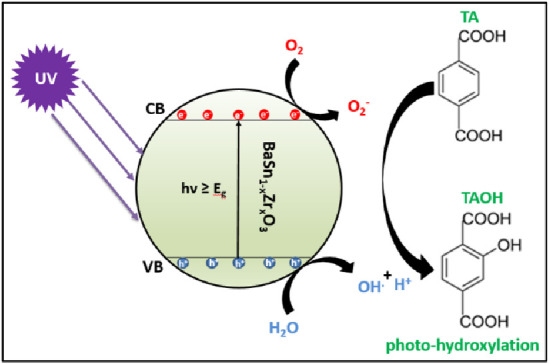
Schematic diagram illustrating the action of BaSn_1–*x*_Zr*_x_*O_3_ as a photocatalyst for the hydroxylation of TA.

**Figure 11:**
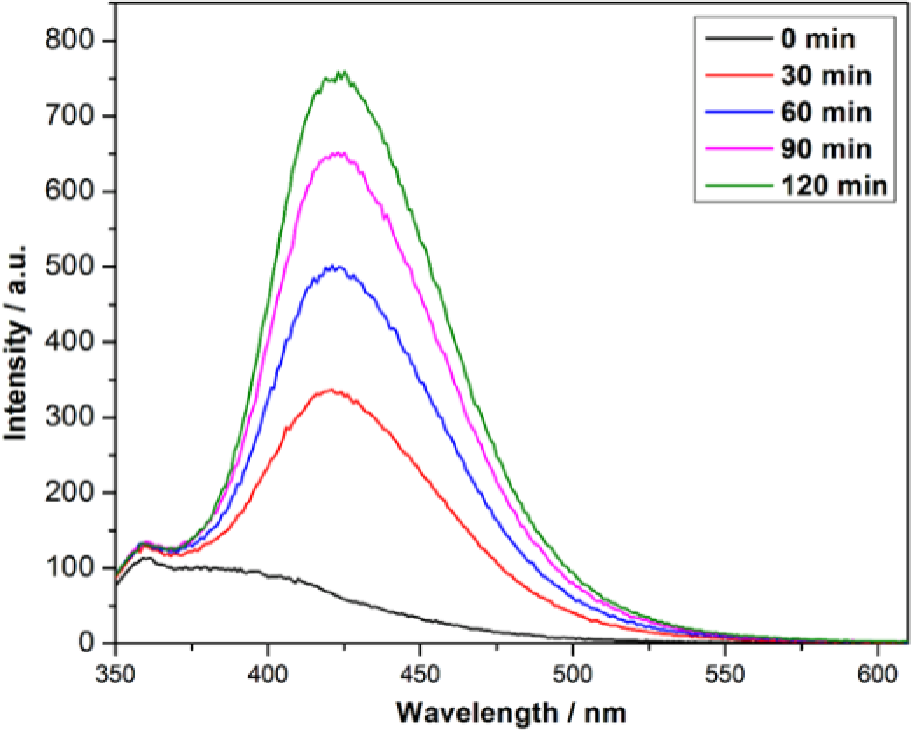
Emission spectra (intensity vs wavelength) as a function of illumination time during the photohydroxylation of terephthalic acid excited at 320 nm on BaSn_0.5_Zr_0.5_O_3_.

**Figure 12:**
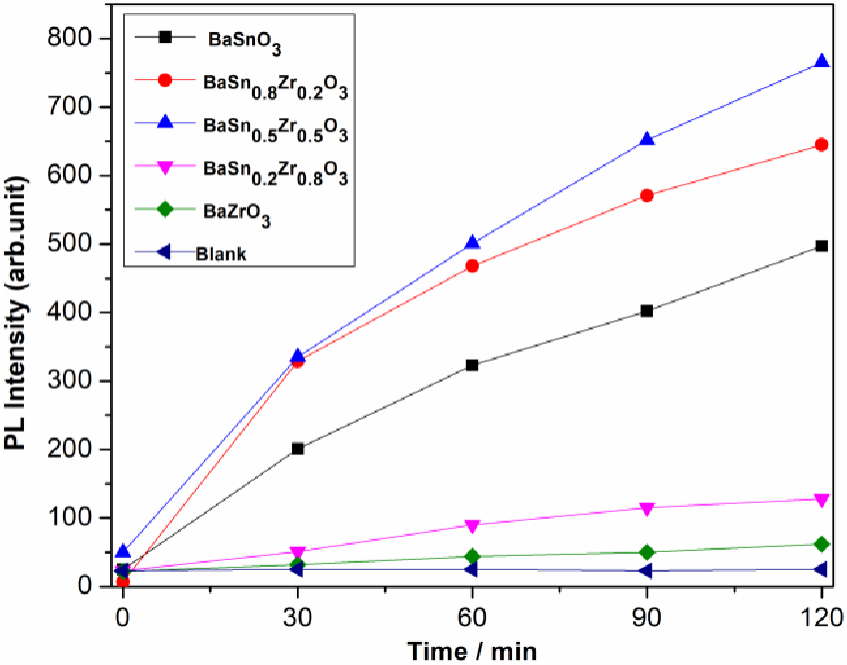
Maximum intensities of fluorescence spectra at 426 nm of TAOH as a function of the irradiation time during the TA hydroxylation.

The photohydroxylation of terephthalic acid to 2-hydroxy terephthalic acid (see Figure 10 for the mechanism)^30^ was used for testing the BaSn_1–*x*_Zr*_x_*O_3_ samples were tested as photocatalysts by monitoring the fluorescence of 2-hydroxy terephthalic acid at 426 nm upon excitation with 320 nm light,^31,32^ see Figure 11 for representative spectra. Figure 12 shows the maximum fluorescence intensity change of TAOH as a function of irradiation time during TA hydroxylation over the different BaSn_1–*x*_Zr_*x*_O_3_samples. It is clear that there is no hydroxylation of TA in the absence of the photocatalyst. The proportionality of the amount of ·OH generated on the surface of BaSn_1–*x*_Zr_*x*_O_3_ to the illumination time can be inferred from the linear relationship between the fluorescence intensity and the irradiation time. However, the photocatalytic activities of BaSn_1–*x*_Zr_*x*_O_3_ samples are diverse and follow the order BaSn_0.5_Zr_0.5_O_3_ > BaSn_0.8_Zr_0._2O_3_ > BaSnO_3_ > BaSn_0.2_Zr_0.8_O_3_ > BaZrO_3_. As can be seen from Figure 12, the BaSn_0.5_Zr_0.5_O_3_ sample with an average crystallite size of 40.25 nm and a specific surface area of 21.52 cm^3^/g exhibited the highest photocatalytic activity compared to the BaZrO_3_ sample with an average crystallite size of 79.04 nm and a specific surface area of 34.18 cm^3^/g. As can be seen from Table 5, the BaSn_0.5_Zr_0.5_O_3_ sample reveals 1.2, 1.5, 6, and 12.3 times higher photo-oxidation potential than BaSn_0.8_Zr_0.2_O_3_, BaSnO_3_, BaSn_0.2_Zr_0.8_O_3_, and BaZrO_3_, respectively. An increase in the OH radical generation with an increase in Zr^4+^ concentration up to *x* = 0.5 can be observed. Further increase in Zr^4+^ leads to a decrease in OH radical generation due to the increase in the band gap and the decreased number of absorbed photons by BaSn_0.2_Zr_0.8_O_3_ and BaZrO_3_. Judging from the PL spectra, the poor activity of BaSn_0.2_Zr_0.8_O_3_ and BaZrO_3_ is related to the low rate of separation of photogenerated electrons and holes. Previous reports have shown that the photocatalytic activity is determined by many factors, including band gap, surface area, particle size, and crystallinity. In general, a suitable band gap, large surface area, high crystallinity, and small crystal size lead to efficient separation of the photogenerated electrons and holes, and thereby improving the photocatalytic activity.^33^ Kudo et al. reported that the particle size affects the charge separation carriers through a decrease of the recombination probability.^34^ Maira et al. noticed that the activity and selectivity of TiO_2_ toward the photo-oxidation of toluene were strongly influenced by the size of particles.^35^ The BaZrO_3_ and BaSn_0.2_Zr_0.8_O_3_ samples show the smallest particle size among all samples but also the lowest photocatalytic performance activity. However, it is expected that a larger surface area creates a large number of reactive sites, leading to enhanced adsorption of reactant.^36^ Thus, there are another factor beside the particle size and band gap that can affect the activity, and our observations show that it is difficult to determine the individual role of every factor and it is rather the combination of several ones. Therefore, the higher activity of BaSn_0.5_Zr_0.5_O_3_ to generate OH radicals compared with that of the other samples can be attributed to the efficient separation of photoinduced carriers as a result of suitable band gap and band structure in addition to the impacts of the increased surface area-to-volume ratio and decreased crystal particle size.

**Table 5:** Fluorescence Signal Intensity of 2-Hydroxyterephthalic Acid (TAOH) at 426 nm

sample	TAOH/a.u.	BET surface area SBET (m^2^/g)
BaSnO_3_	497	4.76
BaSn_0.8_Zr_0.2_O_3_	645	23.99
BaSn_0.5_Zr_0.5_O_3_	765	21.19
BaSn_0.2_Zr_0.8_O_3_	128	36.85
BaZrO_3_	62	34.18

**Figure 13:**
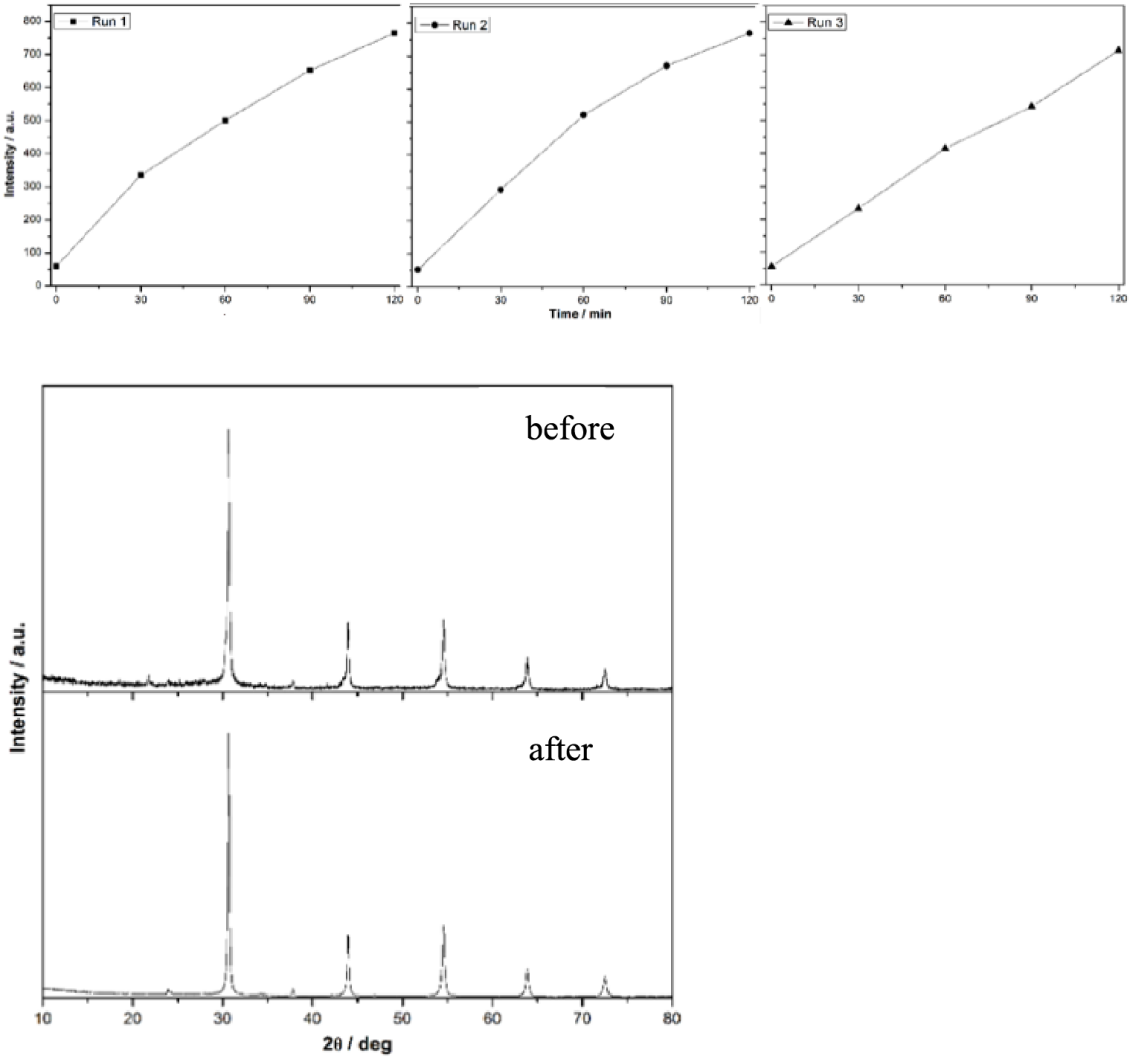
Stability of BaSn_0.5_Zr_0.5_O_3_ after three runs of photohydroxylation of terephthalic acid. Top: intensity change of the 426 nm emission of TAOH upon excitation with 320 nm light. Bottom: PXRD patterns of the BaSn_0.5_Zr_0.5_O_3_ photocatalyst before and after three runs.

Moreover, three photohydroxylation tests of TA over a recycled BaSn_0.5_Zr_0.5_O_3_ sample were performed to check the stability and reusability of the photocatalyst. Figure 13, top, shows the intensity change of the 426 nm emission of TAOH upon excitation with 320 nm light. The photocatalyst was collected after each cycle by centrifugation, washed with deionized water two times, and dried overnight at 80 °C. Furthermore, the weight loss of the photocatalyst during the sampling procedure for each experiment was taken into account, and for each experiment, a fresh TA solution was used. Throughout the cycles BaSn_0.5_Zr_0.5_O_3_ shows a large unaltered activity, pointing to a good stability. Figure 13 (bottom) shows the PXRD patterns of the BaSn_0.5_Zr_0.5_O_3_ sample before and after photohydroxylation, suggesting that during the three cycles, no significant alteration of the structure can be noticed.

## Conclusions

Perovskite barium stannate–zirconate solid solutions were successfully synthesized by microwave irradiation in the ionic liquid [C_4_mim][Tf_2_N] and subsequent low-temperature calcination. This method is a simple and fast, and there is no need to use any additional template or stabilizer, where the ionic liquid plays the role of solvent, microwave absorbance agent, and capping agent. The replacement of Sn in BaSnO_3_ with Zr influences the morphology, crystallinity, and photocatalytic activity. The BaZrO_3_ sample shows the highest crystallinity, while the BaSn_0.2_Zr_0.8_O_3_ sample is the least crystalline material. Based on the SEM measurements, the morphology of the BaSn_1–*x*_Zr_*x*_O_3_ samples varies from rods to spherical with the increase of Zr concentration in the lattice of BaSnO_3_. A diverse catalytic activity of the BaSn_1–*x*_Zr_*x*_O_3_ samples can be noticed toward photohydroxylation of terephthalic acid under UV irradiation. The order of the activities of samples for TA hydroxylation was BaSn_0.5_Zr_0.5_O_3_ > BaSn_0.8_Zr_0.2_O_3_ > BaSnO_3_ > BaSn_0.2_Zr_0.8_O_3_ > BaZrO_3_. The highest photocatalytic performance of BaSn_0.5_Zr_0.5_O_3_ is due to a combination of a suitable band gap and the location of the valence and conduction band associated with the synergistic impacts of the increased surface area-to-volume ratio coupled to the decreased crystal size.

## Supporting Information Available

Synthesis and exploration of barium stannate–zirconate BaSn_1–*x*_Zr_*x*_O_3_ (0 ≤ ≤ 1) solid solutions as photocatalysts. Powder X-ray diffraction patterns, Rietveld refinements, Raman and UV–vis absorption spectra (PDF)
