# Supplementary material for: Synthesis and Exploration of Barium Stannate–Zirconate BaSn1–xZrxO3 (0 ≤ X ≤ 1) Solid Solutions as Photocatalysts
Source: Inorg Chem. 2024 Mar 27;63(14):6132–40. doi: 10.1021/acs.inorgchem.3c02874 (PMC11005035; doi:10.1021/acs.inorgchem.3c02874)

Supporting Information

Synthesis and exploration of barium stannate–zirconate $BaSn_{1-x}Zr_xO_3$ ($0 \leq x \leq 1$) solid solutions as photocatalysts

Tarek Alammar,[a] Anja-Verena Mudring*[b,c]

[a] Department of Chemistry, College of Science, King Faisal University, P.O Box 400 Al-Ahsa 31982, Saudi Arabia.

[b] intelligent Advanced Materials, Department of Biological & Chemical Engineering, Aarhus University, Denmark, anja-verena.mudring@bce.au.dk

[c] Department of Physics, Umeå University, 901 87 Umeå, Sweden, anja-verena.mudring@umu.se.

Table of Contents

SI-1. Rietveld refinement results for $BaSn_{1-x}Zr_xO_3$(x = 0, 0.2, 0.5, 0.8, 1).

SI-2. PXRD pattern of $BaSnO_3$ prepared in demineralized water without ionic liquid and database patterns for $BaCO_3$ and $BaSnO_3$ for comparison.

SI-3. Detailed XPS scans of the Ba3*d*, Sn3*d*, Zr3*d* and O1*s* regions for the various $BaSn_{1-x}Zr_xO_3$ samples.

SI-4. Raman spectra of the respective $BaSn_{1-x}Zr_xO_3$ samples.

SI-5. UV-vis absorption spectra ofthe respective $BaSn_{1-x}Zr_xO_3$ samples.

9 Figures and 5 Tables.

SI-1. Rietveld refinement results for $BaSn_{1-x}Zr_xO_3$(x = 0, 0.2, 0.5, 0.8, 1).

$BaSnO_3$ *Pm*-3*m*

Cell parameters: a = 4.11764(7) Å, b = 4.11764(7) Å, c = 4.11764(7) Å, α = β = γ = 90°

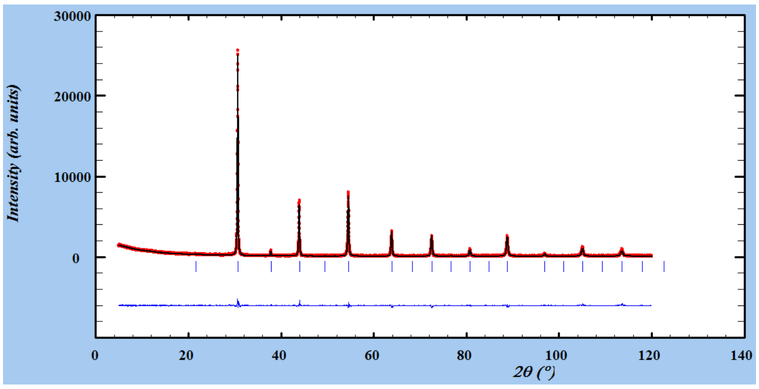

Atom	*Wyckhoff position*	*x*	*y*	*z*	Occupancy
Ba	1b	½	½	½	1
Sn	1a	0	0	0	1
O	3d	½	0	0	1

Rp = 9.75, wRp = 11.4, Rexp = 7.29, χ^2 = 2.43

$BaSn_{0.8}Zr_{0.2}O_3$ *Pm-3m*

Cell parameters: *a* = 4.11947(7) Å, *b* = 4.11947(7) Å, *c* = 4.11947(7) Å, α = β = γ = 90°

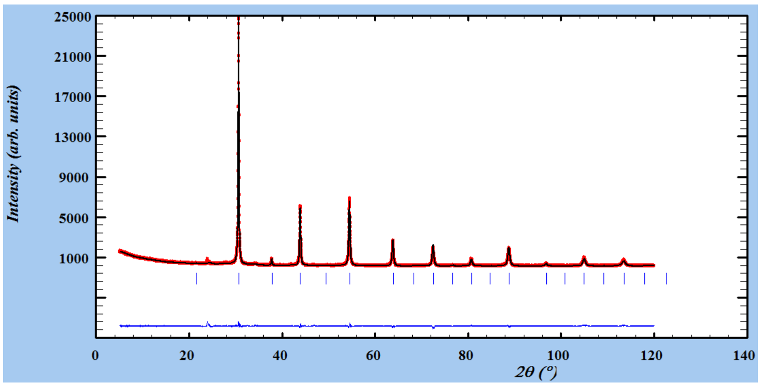

Atom	*Wyckhoff position*	*x*	*y*	*z*	Occupancy
Ba	1b	½	½	½	0.021
Zr	1a	0	0	0	0.004
Sn	1a	0	0	0	0.017
O	3d	½	0	0	0.062

Rp = 8.29, wRp = 9.52, Rexp = 7.30, χ^2 = 1.70

$BaSn_{0.5}Zr_{0.5}O_3$ *Pm*-3*m*

Cell parameters: a = 4.11872(1) Å, b = 4.11872(1) Å, c = 4.11872(1) Å, α = β = γ = 90°

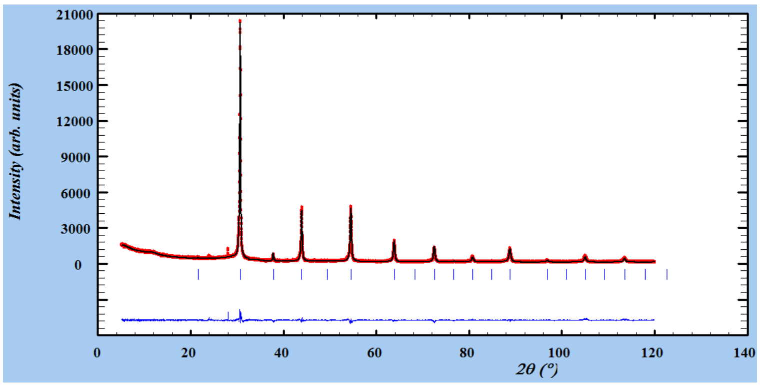

Atom	*Wyckhoff position*	*x*	*y*	*z*	Occupancy
Ba	1b	½	½	½	0.021
Zr	1a	0	0	0	0.010
Sn	1a	0	0	0	0.010
O	3d	½	0	0	0.062

Rp = 12.9, wRp = 13.1, Rexp = 8.65, χ^2 = 2.29

$BaSn_{0.2}Zr_{0.8}O_3$ *Pm*-3*m*

Cell parameters: *a* = 4.17007(2) Å, *b* = 4.17007(2) Å, *c* = 4.17007(2) Å, α = β = γ = 90°

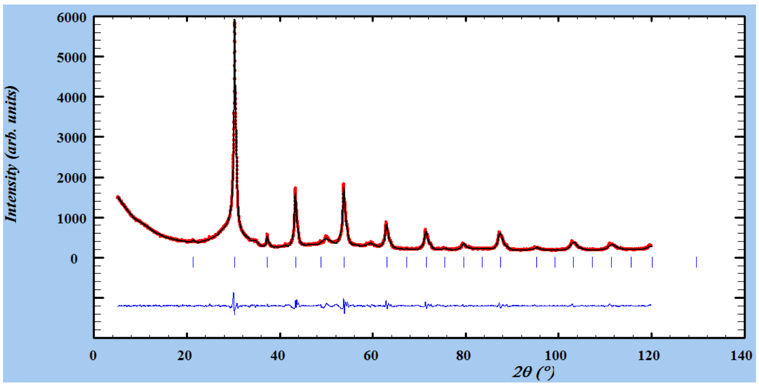

Atom	*Wyckoff position*	*x*	*y*	*z*	Occupancy
Ba	1b	½	½	½	0.021
Zr	1a	0	0	0	0.017
Sn	1a	0	0	0	0.004
O	3d	½	0	0	0.062

Rp = 9.25, wRp = 9.98, Rexp = 10.96, χ^2 = 0.83

$BaZrO_3$ *Pm*-3*m*

Cell parameters: *a* = 4.18794(7) Å, *b* = 4.18794(7) Å, *c* = 4.18794(7) Å, α = β = γ = 90°

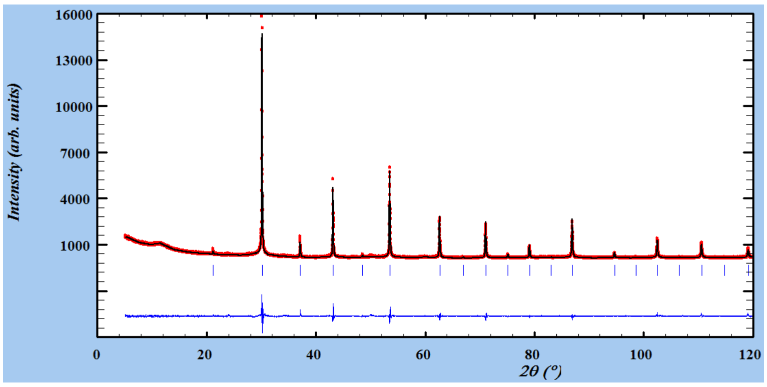

Atom	*Wyckhoff position*	*x*	*y*	*z*	Occupancy
Ba	1b	½	½	½	0.021
Zr	1a	0	0	0	0.021
O	3d	½	0	0	0.062

Rp = 16.5, wRp = 16.1, Rexp = 9.03, χ^2 = 3.19

SI-2. PXRD pattern of $BaSnO_3$ prepared in demineralized water without ionic liquid and database patterns for $BaCO_3$ and $BaSnO_3$ for comparison.

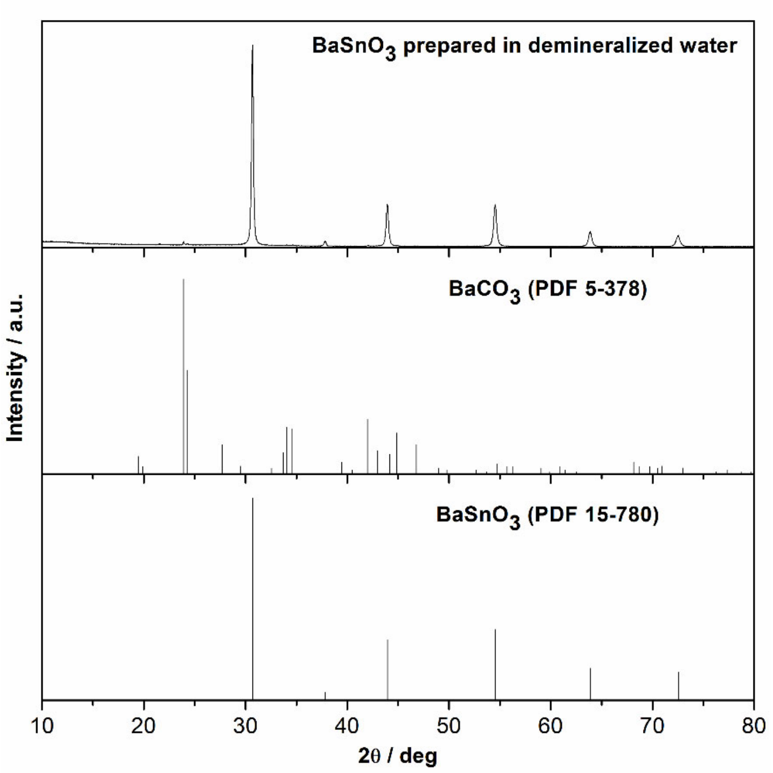

SI-3.

Detailed XPS scans of the Ba3*d*, Sn3*d*, Zr3*d* and O1*s* regions for the various$BaSn_{1-x}Zr_xO_3$ samples.

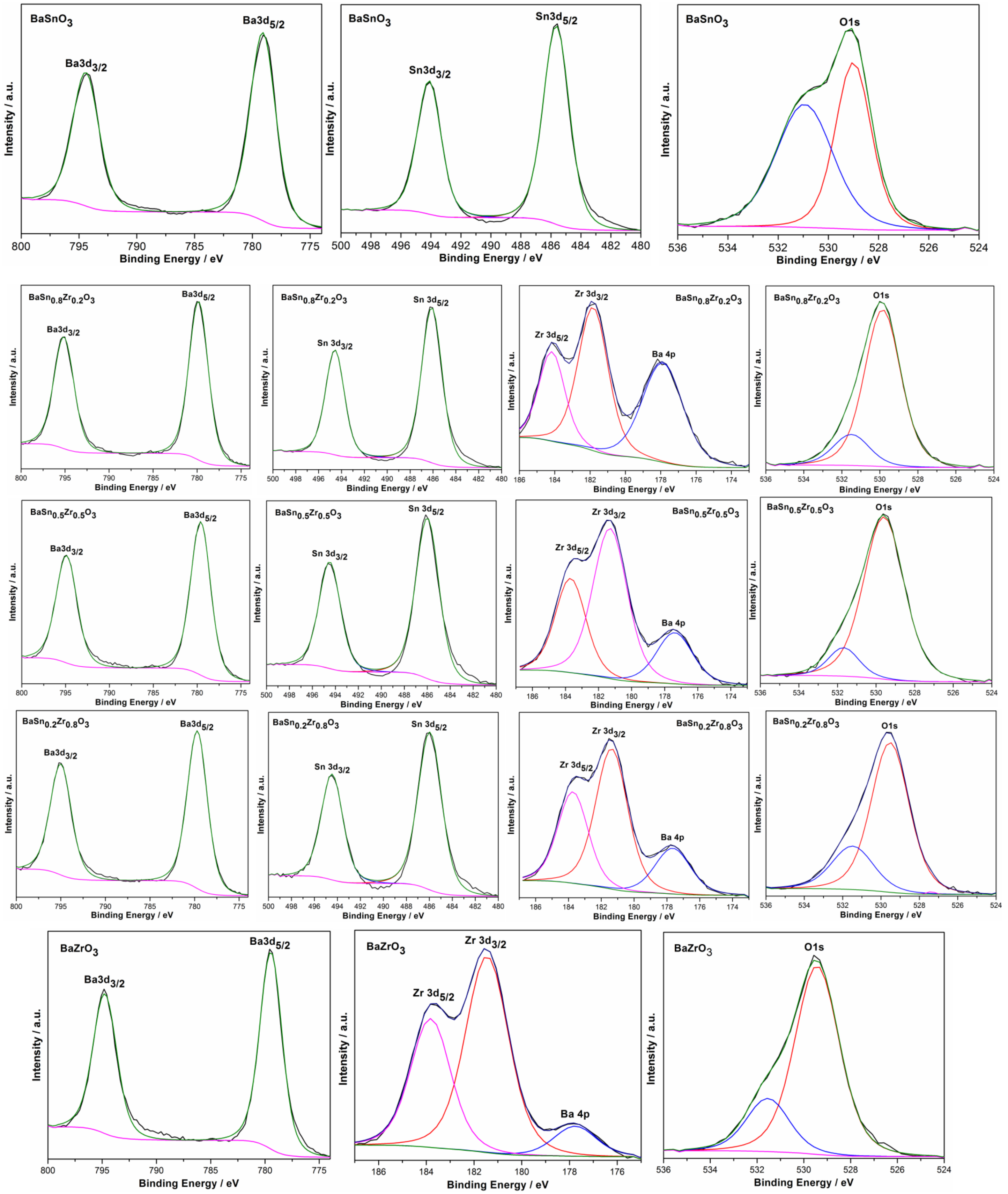

SI-4. Raman spectra of the respective $BaSn_{1-x}Zr_xO_3$ samples.

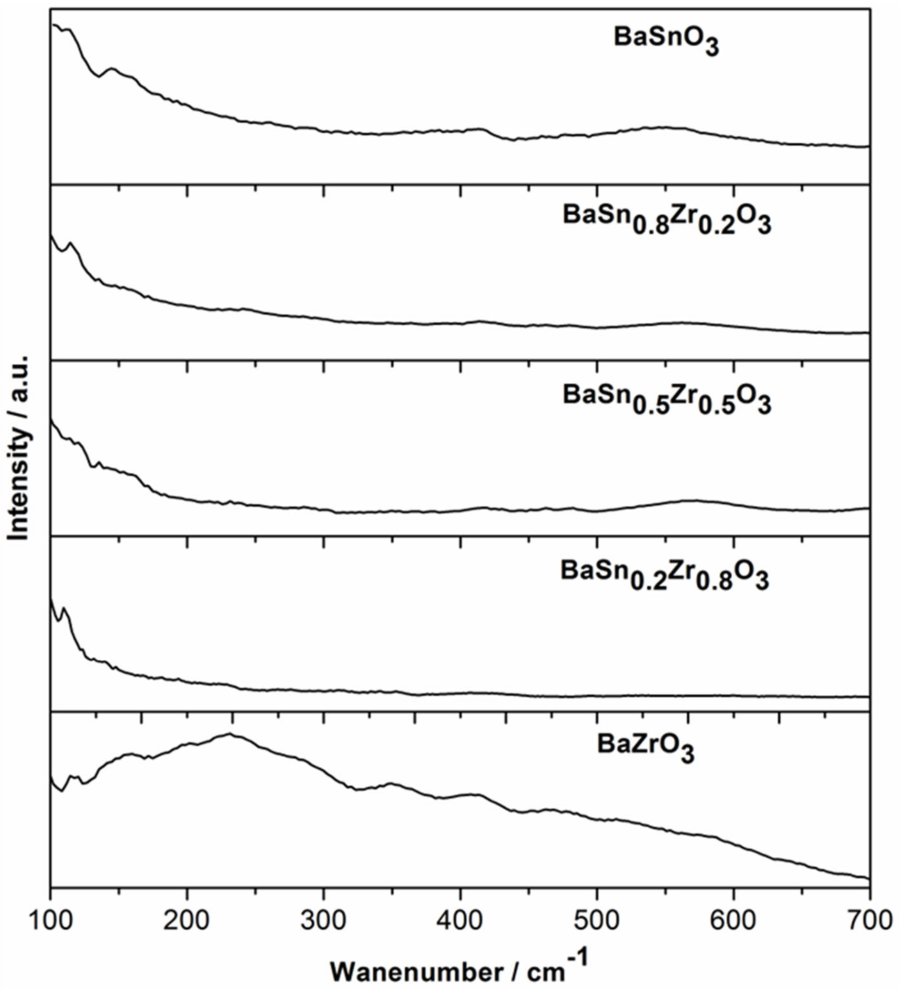

SI-5. UV-vis absorption spectra ofthe respective $BaSn_{1-x}Zr_x O_3$ samples.

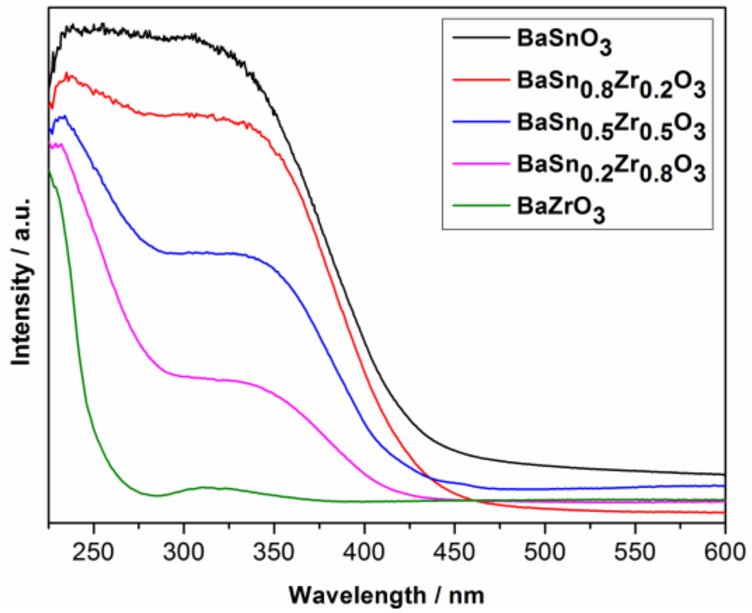

Supplement: Supplementary file 1 — ic3c02874_si_001.pdf [file ic3c02874_si_001.pdf]
